# Effects of a grape-supplemented diet on proliferation and Wnt signaling in the colonic mucosa are greatest for those over age 50 and with high arginine consumption

**DOI:** 10.1186/s12937-015-0050-z

**Published:** 2015-06-19

**Authors:** Randall F. Holcombe, Micaela Martinez, Kestutis Planutis, Marina Planutiene

**Affiliations:** 1 Division of Hematology & Medical Oncology, Tisch Cancer Institute, Icahn School of Medicine at Mount Sinai, One Gustav L. Levy Place, Box 1128, New York, NY 10029 USA; 2 University of California, Irvine, USA

**Keywords:** Diet, Cancer prevention, Resveratrol, Colon cancer, Aging, Arginine

## Abstract

A diet rich in fruits and vegetables, and a grape-derived compound, resveratrol, have been linked to a reduced incidence of colon cancer. In vitro and in vivo, resveratrol suppresses Wnt signaling, a pathway constitutively activated in over 85 % of colon cancers.

Thirty participants were placed on a low resveratrol diet and subsequently allocated to one of three groups ingesting 1/3-to-1 lb (0.15–0.45 kg) of grapes per day for 2 weeks. Dietary information was collected via 24-h recall. Colon biopsies for biomarker analysis were obtained pre- and post-grape and evaluated for the expression of Wnt pathway target genes and for markers of proliferation by RT-PCR and immunohistochemistry.

Participants lost an average of 2 · 6 lb (1.2 kg, *p* = 0 · 0018) during the period of grape ingestion. The expression of CyclinD1 (p < 0 · 01), AXIN2, CD133 (*p* = 0 · 02) and Ki67 (*p* = 0 · 002) were all reduced after grape ingestion. Individuals over 50 years of age and those with high dietary arginine consumption had increased basal expression of CyclinD1, AXIN2, cMYC and CD133 (p value range 0 · 04 to <0 · 001) that, following grape ingestion, were reduced to levels seen in younger participants.

The reduction in Wnt signaling and mucosal proliferation seen following short-term ingestion of 1/3–1 lb (0.15–0.45 kg) of grapes per day may reduce the risk of mutational events that can facilitate colon carcinogenesis. The potential benefit is most marked for high-risk older individuals and individuals whose diet is high in arginine intake. Dietary grape supplementation may play a role in colon cancer prevention for high-risk individuals.

## Introduction

Studies suggest strongly that a diet rich in fruits and vegetables leads to a lower risk of colorectal cancer (CRC) [1, 2]. Grape seeds and other grape-based products have purported CRC prevention activity [3], are rich in polyphenols and contain resveratrol, anthocyanins, catechins, quercetin and numerous other compounds with chemopreventive potential [4]. The most intensively studied component in grapes is resveratrol which suppresses PI3-kinase, AKT and NF-kB signaling pathways [5] and may affect tumor growth by a myriad of other mechanisms as well [6, 7]. Systemic administration of resveratrol has been shown to inhibit the growth of intestinal tumors in several different rodent cancer models [8, 9]. For colon cancer prevention, effects are seen over a wide variety of dose ranges depending on individual studies. Tessotore [10] demonstrated activity of very low dose resveratrol of 0.2 mg/kg/day in reducing aberrant crypt foci (ACF) in the colon in an azoymethane-induced tumor model. In another carcinogen-based model, utilizing 1,2-dimethylhydrazine, resveratrol at 8 mg/kg/day reduced both ACF and colonic tumors [11] as did gavage of 60 mg/kg body weight [12]. In genetic models utilizing the APCmin/+ mouse, which harbors a single allele mutation in *apc* and therefore has intrinsically activated Wnt signaling, Schneider [13] demonstrated profound activity at dosages as low as 0.3 mg/mouse/day in reducing intestinal tumors. In this study, expression of Wnt target gene cyclinD1 as well as other markers of cell cycling was reduced.

Resveratrol, even at low concentrations, blocks Wnt signaling in colon cancer cells in vitro [14]. This pathway is activated in over 85 % of CRC making it an attractive target for a colon cancer prevention agent. Resveratrol-rich freeze-dried grape powder has been utilized in a pilot study in normal human volunteers and was found to down-regulate the expression of Wnt pathway target genes CyclinD1 and AXIN2 in colonic mucosa [15]. However, low bioavailability of individual compounds such as resveratrol often results in systemic concentrations too low to be clinically active [16]. Therefore, it is important in consideration of dietary approaches to cancer prevention to consider the aggregate activity of all of the bioactive components in a particular foodstuff [17] and not just single purified compounds.

This phase I study was undertaken to evaluate the potential role of a grape-supplemented diet for CRC prevention. The endpoints were biologic biomarkers of proliferation and Wnt signaling in colonic mucosa. During the study, detailed dietary information was collected and analyzed. This study is unique in that the effects of the complete foodstuff, rather than a refined component (ie grape seed extract) or individual substance (ie resveratrol) on biologically relevant cancer prevention endpoints is being investigated.

## Materials and methods

### Clinical trial design and conduct

**Fig. 1:**
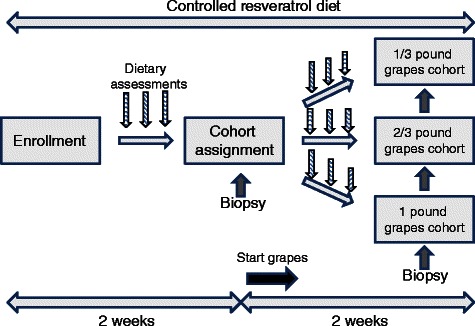
Schematic of the clinical trial design. The controlled reservatrol diet was maintained throughout the 4 week period. Grape ingestion occurred during the final 2 weeks

30 healthy non-diabetic volunteers were enrolled over a period of 9 months for this study (clinical trial NCT00578396) which was approved by the University of California, Irvine institutional review board (ethics committee). Written informed consent was obtained for all participants. One potential subject was excluded from the study because of an elevated blood sugar discovered during eligibility testing that included a complete blood count and chemistry profile. This individual was referred for evaluation of occult diabetes. All participants had an initial consultation with a dietitian and were placed onto a low resveratrol diet (no grapes, wine, raisins, peanuts, peanut butter or cranberries) for 4 weeks. The controlled resveratrol diet was instituted as a base diet prior to sample acquisition and continued during the phase of grape ingestion. This was done to ensure minimal impact of potential dietary changes during the interventional component of the study. 24 h dietary recall evaluations were performed 3 times during days 1–14, and three more times during days 15–30, with data entered into a dietary software program “Nutrition Data System for Research (NDSR) Versions 2006–2008” produced by the University of Minnesota. During days 15–30, participants were assigned to one of three grape consumption cohorts: 1/3 lb, 2/3 lb or 1 lb (0.15 kg, 0.30 kg, 0.45 kg) of grapes to be ingested each day. These amounts were selected by the investigators because 1/3 lb of grapes is equivalent to one “serving” as defined by the United States Department of Agriculture (USDA). Each patient was given a voucher redeemable at a local grocery store for red, seedless grapes and a digital kitchen scale. Compliance with grape consumption was ascertained at the time of the 24 h recall evaluations and again at the end of subject participation. Assignment into the cohorts was determined by a random sequential enrollment algorithm. Limited flexible sigmoidoscopies were performed on day 15 (“pre-grape”) and on day 30 (“post-grape”). Two-to-four rectal mucosal biopsies were obtained for RNA and immunohistochemical analysis. See Fig. 1 for a schematic of the clinical trial design.

### 24 hour dietary recall

Nutrition data system for research (NDSR) is a dietary analysis program designed for the collection and analyses of 24-h dietary recalls and the analysis of food records, menus, and recipes. Nutrient intake from both food and supplemental sources are captured and quantified. Dietary interviews took place over the telephone and/or in person and data was entered directly into NDSR. Food portion estimation visual aids and a kitchen scale were provided to respondents to assist in portion size estimation.

### Quantitative Real-time PCR (qRT-PCR)

RNA was extracted from one-to-two of the biopsies obtained from each of the “pre-grape” and “post-grape” sigmoidoscopies using standard methodologies. Rectal mucosal biopsies were analyzed by quantitative real-time PCR for the expression of genes associated with proliferation and Wnt signaling including: ornithine decarboxylase (ODC1), nemo-like kinase (NLK), cJUN, cMYC, cyclinD1 (CCND1), lymphoid enhancing factor-1 (LEF1, TCF7L3), axinII (AXIN2), forkhead box protein 3 (FOXO3) and CD133 (Prominin1, PROM1). Primer pairs were obtained from Qiagen (Valencia, CA) with cycling parameters as defined by the manufacturer. Wnt target gene mRNA levels were all normalized to the housekeeping gene ribosomal RNA (rRNA). The relative RNA expression was calculated by the comparative threshold cycle method. All experiments were repeated in triplicate.

### Immunohistochemical analysis

One-to-two of the biopsies obtained from each of the “pre-grape” and “post-grape” sigmoidoscopies were immediately placed into 10 % formalin for processing and subsequently paraffin embedded, sectioned, and placed onto slides for Ki67 immunohistochemistry (IHC). The percentage of cells expressing Ki67 in the lower 1/3 of colonic crypts was determined by fluorescence confocal microscopy. A minimum of 50 crypts were evaluated for each set of biopsies.

### Statistical analysis

Patient characteristics were compared with 1-way ANOVA with a Newman-Keuls post-test. Dietary changes seen with grape supplementation, pre- and post-grape gene expression by qRT-PCR and Ki67 expression scored by IHC were analyzed with a Wilcoxon matched pairs signed-rank test. Linear regression analysis was utilized to generate r^2^ and p values for the comparison of AXIN2 and CCND1 expression. Comparison of expression levels stratified for age or for dietary arginine intake was undertaken with an unpaired *t*-test. Statistical significance was defined at a level of p < 0 · 05.

## Results

### Clinical trial and dietary analysis results

There were no statistically significant differences between the any of the three cohorts of grape consumption for age, initial weight or body mass index (BMI; Table 1). Results related to dietary changes were not statistically different across the three cohorts and therefore data were combined for presentation in this report. More females participated in the study. The race/ethnicity of the subjects is depicted in Table 1. No toxicities were reported for any of the 30 participants in the clinical trial. Toxicities were monitored by detailed interviews by research personnel. Compliance with the assigned grape consumption daily ingestion was 100 % for the two week period being monitored. All participants said they were compliant with the dietary restrictions to limit resveratrol-containing foods for the entire 4 week study period.

**Table 1:** Participant characteristics

	Overall group (mean ± SE)	1 pound cohort (mean ± SE)	2 pound cohort (mean ± SE)	3 pound cohort (mean ± SE)	Significance (p)*
Age	43 · 28 ± 2 · 28	46 · 00 ± 3 · 02	43 · 45 ± 4 · 79	40 · 10 ± 3 · 98	NS
Initial weight (lb)	172 · 1 ± 6 · 55	175 · 3 ± 16 · 30	171 · 4 ± 8 · 22	169 · 2 ± 7 · 67	NS
BMI	28 · 12 ± 1 · 07	27 · 89 ± 2 · 42	27 · 58 ± 1 · 63	29 · 01 ± 1 · 09	NS
Gender (#)					
Male	9	6	2	1	
Female	21	4	8	9	
Total	30	10	10	10	
Race/Ethnicity (#)					
White	18	5	7	6	
Black	2	1	1	0	
Chinese	2	2	0	0	
Filipino	1	1	0	0	
Hispanic	4	0	0	4	
Japanese	1	0	1	0	
Korean	1	0	1	0	
Persian	1	1	0	0	
Total	30	10	10	10	

*BMI* Body Mass Index

*1-way Anova with Newman-Keuls post test

**Fig. 2:**
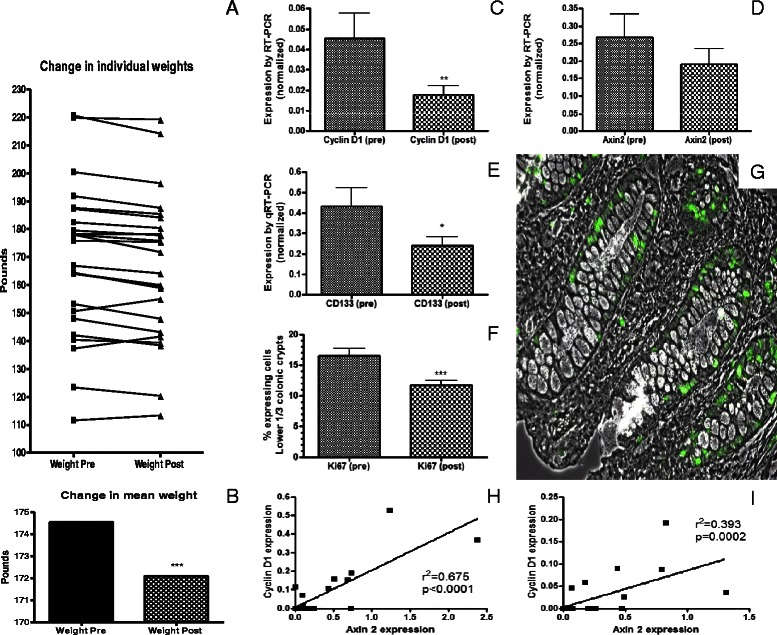
Change in individual (**a**) and mean (**b**) weights for the 30 participants from pre-grape ingestion timepoint to the post-grape ingestion timepoint. Grape ingestion significantly reduced expression as measured by qRT-PCR of CyclinD1 (**c**) and CD133 (**e**) with a non-statistically significant reduction in AXIN2 (**d**). However, CyclinD1 and AXIN2 levels in colonic mucosa were strongly correlated with each other both pre-grape ingestion (**h**) and post-grape ingestion (**i**). Ki67 expression was also reduced following grape ingestion (**f**). This was measured by calculating the percentage of positively staining cells in the lower 1/3 of colonic crypts (**g**; representative photomicrograph—green represents positive immunofluorescence). *p < 0 · 05; ** p < 0 · 01; *** p < 0 · 005

During the two weeks of grape consumption, participants lost an average of 2 · 6 lbs (1 · 2 Kg; *p* = 0 · 0018; Fig. 2) despite significant increases in total carbohydrates, percent of calories from carbohydrates and total sugars (Table 2). Overall KCALS were unchanged. Participants reported that they often felt “full”, particularly those consuming 1 lb (0.45 kg) of grapes per day, and subjectively reported that they snacked less because of this. Interestingly, there was a significant reduction in the percentage of calories from fat (35 · 32 to 30 · 77 %) while ingesting grapes (*p* = 0 · 011; Table 2). Other significant changes included an increase in copper, potassium and vitamin B6 and a decrease in Biochanin A ingestion.

**Table 2:** Dietary changes observed with grape supplementation

Parameter	Pre-grape baseline	At conclusion of grape intervention	Direction of change	Significance (p)*
Weight (lbs)	172 · 1 ± 6 · 55	170 · 7 ± 7 · 65	↓	0 · 0018
KCALS	1960 ± 131 · 2	1984 ± 125 · 1	↔	NS
Carbohydrate (gm)	233 · 4 ± 15 · 8	271 · 3 ± 18 · 2	↑	0 · 0048
Calories from carbohydrate (%)	46 · 99 ± 1 · 58	51 · 87 ± 1 · 53	↑	0 · 0089
Total sugars (gm)	92 · 49 ± 7 · 86	131 · 9 ± 9 · 44	↑	0 · 0001
Calories from fat (%)	35 · 32 ± 1 · 44	30 · 77 ± 1 · 20	↓	0 · 011
Copper (mg)	1 · 32 ± 0 · 13	1 · 53 ± 0 · 11	↑	0 · 0035
Potassium (mg)	2566 ± 189	3004 ± 203	↑	0 · 0014
Vitamin B6 (mg)	1 · 957 ± 0 · 16	2 · 254 ± 0 · 21	↑	0 · 0193
Biochanin A (mg)	0 · 133 ± 0 · 07	0 · 013 ± 0 · 01	↓	0 · 0067
Folate (mcg)	486 · 2 ± 41 · 8	433 · 0 ± 41 · 1	↔	NS
Cholesterol (mg)	289 · 3 ± 34 · 93	262 · 0 ± 28 · 06	↔	NS
Genistein (mg)	2 · 53 ± 1 · 11	0 · 61 ± 0 · 20	↔	NS
Lycopene (mcg)	4798 ± 726	4064 ± 702	↔	NS
Arginine (gm)	4 · 503 ± 0 · 41	4 · 276 ± 0 · 34	↔	NS

Dietary components listed as amount ingested per day

*Wilcoxon matched pairs signed rank test

### Changes in markers of proliferation and Wnt signaling

Results related to changes in proliferation and Wnt signaling markers were not statistically different across the three cohorts. In general the magnitude of the changes was slightly greater in the 1 lb/day (0.45 kg) cohort than the 2/3 lb/day (0.30 kg) and 1/3 lb/day (0.15 kg) cohorts but the direction of the changes were consistent throughout all 3 groups. Each cohort contained only ten participants and therefore data for all participants were combined for presentation in this report. RT-PCR analysis revealed a significant reduction in the expression of CyclinD1, a proliferation marker and target gene for Wnt signaling, following grape ingestion (Fig. 2; p < 0.01). A trend toward reduction in another Wnt target gene, AXIN2 was seen and the pre- and post-grape expression levels of CyclinD1 correlated significantly with the corresponding levels of AXIN2 (Fig. 1). The expression of CD133, a colonic stem cell marker was also significantly reduced (*p* = 0 · 02). The expression of Ki67 protein, as measured by the percentage of expressing cells in the lower 1/3 of colonic crypts, was significantly reduced following grape ingestion (*p* = 0 · 002; Fig. 2). While the expression of several of the other genes tested had consistent trends, none, including cMYC, reached statistical significance.

### Effect of age and grape diet on markers of proliferation and Wnt signaling

**Fig. 3:**
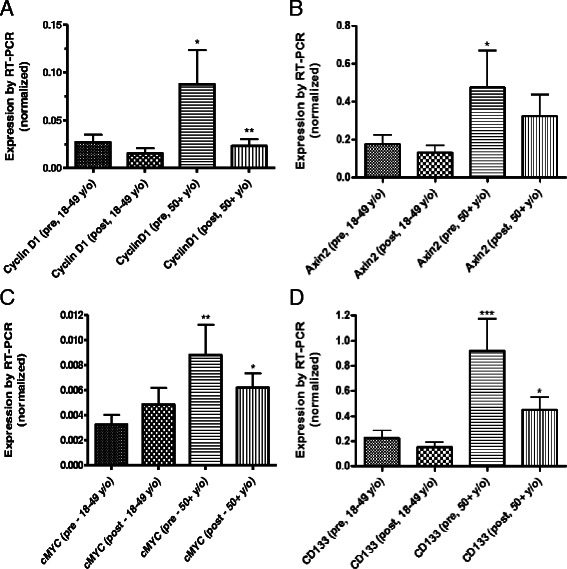
Differences in expression of CyclinD1 (**a**), AXIN2 (**b**), cMYC (**c**) and CD133 (**d**) in participants below age 50 and 50 years old and above. Prior to grape ingestion, older individuals had significantly higher levels of CyclinD1, AXIN2, cMYC and CD133 than individuals aged 18–49. Grape ingestion in participants aged 50 and above led to a reduction in the expression CyclinD1 (p < 0 · 01), AXIN2, cMYC and CD133. Levels of CyclinD1 and AXIN2 in participants aged 50 and above were reduced to levels seen prior to grape ingestion in 18–49 year olds. While reduced after grape ingestion, the level of expression of cMYC and CD133 in participants aged 50 and above remained significantly elevated when compared to the pre-grape ingestion levels of 18–49 year olds. *p < 0 · 05; **p < 0 · 01; ***p < 0 · 001

Data across all cohorts was stratified by age of participant. Twenty one participants were aged 18–49 and 9 were aged 50 and over. Several markers of Wnt signaling and proliferation were very significantly increased in older participants including CyclinD1 (*p* = 0 · 022), AXIN2 (*p* = 0 · 040) and cMYC (*p* = 0 · 006). The stem cell marker CD133 also had significantly increased expression in older individuals (p < 0 · 001; Fig. 3). As can be seen in Fig. 2, virtually all of the effect of grape ingestion was on the older participants, with no change in expression seen for any of these four markers in the 18–49 year old group and significant reductions seen in the 50 and older group. In most cases, the effect of grape ingestion was to reduce the levels of expression of these markers to a level seen basally in the younger participants (difference between “pre-younger” and “post-older” not significant in all cases).

### Effect of arginine intake and grape diet on markers of proliferation and Wnt signaling

**Fig. 4:**
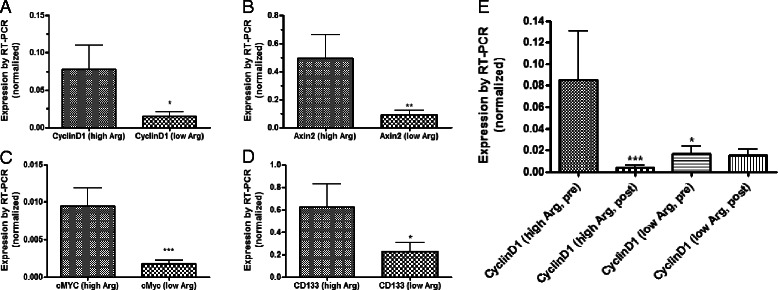
Differences in expression of CyclinD1 (**a**), AXIN2 (**b**), cMYC (**c**) and CD133 (**d**) in participants with high baseline arginine consumption and those with low baseline arginine consumption. Individuals with high arginine intake had significantly higher expression of each of these markers. In those with high arginine intake, grape ingestion led to a statistically significant reduction in CyclinD1 expression to levels seen in those with low arginine intake. Grape intervention did not significantly change the expression of CyclinD1 in participants with low initial arginine intake. *P < 0 · 05; **p < 0 · 01; ***p < 0 · 005

Participants were also stratified according to baseline arginine intake. The mean daily arginine intake was 4 · 5gm/day. Twenty one participants had arginine ingestion below the mean and nine participants had arginine ingestion above the mean. Expression of CyclinD1, AXIN2, cMYC and CD133 were all significantly higher in patients with high baseline arginine intake compared to those with below average arginine intake (p values ranging from 0 · 04 to 0 · 002; Fig. 4). Grape ingestion resulted in a significant reduction in CyclinD1expression in volunteers with high baseline dietary arginine consumption (P < 0 · 0039), achieving expression levels below the baseline CyclinD1 levels for patients in the low arginine group. Reductions following grape ingestion were also seen with cMYC, Axin2 and CD133 but these were not statistically significant.

## Discussion

Participants were able to tolerate up to a pound of grapes per day for 2 weeks with full compliance and without any adverse consequences. No patients with diabetes were enrolled on the trial based on a recommendation from the Institutional Review Board (Ethics Committee) because of the high sugar content of grapes. While on study, participants ingested less fat and lost, on average, 1 · 2 kg of weight. While weight loss with grapes has been reported previously in the popular press [18], to our knowledge this is the first confirmation of this effect under controlled clinical trial conditions.

The effects of a grape supplemented diet on Wnt signaling and markers of proliferation were consistent with prior in vitro [14] studies using resveratrol and an in vivo [15] study using resveratrol supplements and freeze-dried grape powder. Inhibition of Wnt signaling and reduced colonic mucosal proliferation suggests potential cancer preventative effects of a grape-containing diet. Whether these effects are due to resveratrol alone or other constituents of grapes, or a combination of constituents, remains to be defined. This study was of short duration and did not address long-term effects on the appearance of aberrant crypt foci or pre-cancerous colonic adenomas. The significant changes noted after a short period of exposure suggests that intermittent consumption of grapes may be sufficient and this should be considered in the design of future dietary-based cancer prevention trials.

As the human colon ages, the inflammatory process leading to mucosal injury and the regenerative capacity of the epithelium are affected [19]. Telomere shortening, methylation of mucosal healing-associated genes, and alterations of growth factor signaling occur and have been postulated to affect the regenerative capacity of the epithelium. In rats, an increase in the proliferation rate of colonic mucosa is seen in conjunction with senescence [20]. Diet restriction increases intestinal apoptosis in aging rats, perhaps providing protection from an age-related accumulation of DNA alterations [21]. Resveratrol has been shown to improve survival of mice on a high calorie diet [22], possibly through its effects on suirtuin-1 (SIRT1) activation [23]. Indeed, small molecule SIRT1 activators have been proposed for the treatment of age-related disorders [24]. In our study, colonic proliferation was significantly higher in older participants compared to younger participants. The effects of grape ingestion were most dramatically seen in the older population, with a reduction of both Wnt target genes and markers of mucosal proliferation.

High levels of dietary arginine and the polyamine synthesis pathway have been linked to colon carcinogenesis in several types of animal models [25, 26]. This has also been implicated in human colon carcinogenesis [27] and a randomized trial has demonstrated that an inhibitor of polyamine synthesis, DFMO, reduces the incidence of colon adenomas with high malignant potential [28]. Ornithine decarboxylase (ODC) catalyzes the rate-limiting step in the biosynthesis of polyamines and is inhibited by resveratrol, suggesting that one of the molecular mechanisms through which resveratrol may be operating is polyamine pathway inhibition [29]. In our study, dietary arginine consumption was ascertained through the diet survey instrument. When stratified for arginine consumption, the effect on colonic mucosal proliferation was most marked in participants who had higher levels of arginine ingestion. Based on the ongoing dietary monitoring and post-grape intervention data, this effect appears to have been due to the grape consumption and not due to a reduction in arginine consumption over the course of the study.

This study has significant implications for colon cancer prevention. The reduction in Wnt signaling and mucosal proliferation seen following relatively short-term ingestion of 1/3–1 lb of grapes should reduce the risk for the development of mutational events that can ultimately result in colon carcinogenesis. The potential benefit is most marked for older individuals and individuals whose diet is high in arginine intake. Both of these groups have an increased incidence of colon cancer [27, 30]. Subsequent studies should focus on other high-risk populations such as patients with a history of colonic adenomas (polyps) and individuals with an inherited genetic predisposition for colon cancer. Finally, patients with inflammatory bowel disease may benefit from the anti-proliferative effects of a grape-supplemented diet.
